# Midwifery care in The Gambia: A focus group study with clinical midwives, midwifery students, educators and leaders on how barriers and facilitators impact quality midwifery care

**DOI:** 10.1371/journal.pone.0318304

**Published:** 2025-02-07

**Authors:** Lamin Suwareh, Helena Lindgren, Kerstin Erlandsson, Haddy Tunkara Bah, Evelina Holm, Majda Meljoum, Omar Manjang, Saineh Sanneh, Baboucarr Cham, Ulrika Byrskog

**Affiliations:** 1 School of Nursing and Midwifery, The Gambia College, Banjul, The Gambia; 2 Department of Health Promotion, Sophiahemmet University, Stockholm, Sweden; 3 Department of Women’s and Children’s Health, Karolinska Institute, Stockholm, Sweden; 4 School of Health and Welfare, Dalarna University, Falun, Sweden; 5 School of Medicine & Allied Health Sciences, University of The Gambia, Banjul, The Gambia; 6 Ministry of Health, Banjul, The Gambia; Jhpiego, UNITED STATES OF AMERICA

## Abstract

**Objective:**

The aim of this study is to explore the impact of barriers and facilitators on the quality of midwifery care in The Gambia, from the perspectives of clinical midwives, midwifery students, educators, and leaders.

**Methods:**

A qualitative study based on focus group discussions with 29 clinical midwives, midwifery students, educators and leaders analysed with content analysis. The study was conducted in The Gambia.

**Results:**

The analyses led to three main categories outlining barriers and facilitators for the quality of midwifery care: 1) *the gap between theory and practice, 2) working in a harsh environment and 3) facilitating factors that can pave ways forward.* The results are described in generic categories: 1a) national plans and facility-based guidelines, 1b) midwifery education, 1c) becoming a skilled midwife, 2a) scarcity of resources, 2b) encountering community barriers, 2c) midwives - a passionate but demotivated profession, 3a) positive assets for quality midwifery care, 3b) women in leadership as a tool for a motivated midwifery workforce and 3c) teamwork.

**Conclusions:**

Addressing the gaps between theory and practice, and strengthening the incentives for midwives to remain in their profession are central for improved quality of midwifery care in The Gambia. Guaranteed employment after completing education, equal opportunities for men and women to become midwives and the significance of passion are assets which need to be carefully maintained within the health care system.

## Introduction

In The Gambia, reproductive and newborn health services are primarily provided by professional midwives who operate autonomously within the healthcare system. The midwifery workforce includes individuals with various levels of education, such as Registered Midwives (RMs), State Enrolled Midwives (SEMs), and Enrolled Community Health Midwives (ECHMs). These professionals are responsible for delivering essential care throughout pregnancy, childbirth, and the postpartum period [1]. The national skilled health worker density is 1.55 per 1,000 population, and the nurse midwife density is 0.27 per 1,000 population. Both figures fall significantly short of the World Health Organization’s (WHO) recommended density threshold of 4.45 per 1,000 population for each health cadre [2]. In addition to the presence of limited midwifery professionals, The Gambia continues to face substantial maternal and neonatal health challenges. The maternal mortality ratio remains high at 289 per 100,000 births, with a neonatal mortality ratio of 29 per 1,000 births and a fertility rate of 4.4 births per woman. The caesarean section rate is low at 2.0%, and although 84% of births are attended by skilled personnel, the under-five mortality rate persists at 48 per 1,000 births [3–5].

Aligned with the Sustainable Development Goals (SDGs), which aim to reduce maternal mortality to 70 per 100,000 live births by 2030, significant efforts are needed to strengthen the midwifery workforce and enhance skilled attendance at births [6]. This requires improving midwifery education and ensuring that midwives have up-to-date knowledge and skills in sexual and reproductive health (SRH) [7]. However, gender inequality and the low socio-cultural status of midwifery present significant challenges. Often perceived as “women’s work,” midwifery lacks recognition and is subject to power imbalances within the healthcare system [8]. This not only causes moral distress and burnout among midwives but also compromises the quality of care provided to women and newborns, perpetuating poor health outcomes and human rights violations [8]. Evidence suggests that midwife-led care for low-risk women during labor results in better outcomes and fewer interventions compared to physician-led care [6,9]. Realizing the potential of midwife-led care, however, requires that midwives are equipped with the necessary skills, work in supportive environments, and are backed by proactive leadership at both facility and national levels [6].

Despite the presence of midwifery professionals in The Gambia, high rates of maternal and neonatal mortality persist. Power imbalances, inadequate education, limited human and material resources, lack of continuity of care and support during birth, and high attrition rates are described [2,10–13]. Addressing these challenges is crucial for achieving the SDGs and improving maternal and neonatal health outcomes in The Gambia. By engaging midwifery stakeholders in different positions in the country, this research seeks to inform strategies for strengthening the midwifery workforce and improving the quality of care delivered to women and newborns. The aim of this study is to explore the impact of barriers and facilitators on the quality of midwifery care in The Gambia, from the perspectives of clinical midwives, midwifery students, educators, and leaders.

## Materials and methods

An empirical qualitative design, with focus group discussions (FGDs) [14] analysed by inductive content analysis [15], was applied in this study.

### Recruitment and participants

A purposive sampling method was employed [14,16]. Recruitment took place between April 21st and 27th, 2023, led by the local project coordinator, a former head of staff at one of the participating institutes with extensive local and national expertise in the relevant field. The coordinator strategically invited participants from various sectors within the midwifery profession and from different regions of The Gambia. This approach ensured a broad range of perspectives from individuals with experience and knowledge in midwifery, who could provide valuable insights into barriers and opportunities (Box 1). The clinical midwives, midwifery students and midwifery leaders were selected from West Coast Regions I and II where about 79% of the Human Resource for Health (HRH) are located [2]. To achieve nationwide representation, the midwife educators were selected from the country’s five midwifery education institutions throughout the country; Department of Nursing and Reproductive Health University of the Gambia, The Gambia College School of Nursing and Midwifery; The American International University, and the two State Enrolled Nursing and Midwifery, and Enrolled Community Health Nurses and Midwives education schools. In order to be included in the study, inclusion criteria were 1) having at least one year of work experience in the professional role as a midwife, except for the midwifery students who needed to have reached their final semester, 2) currently being actively working or studying midwifery. Students who had not reached the last semester were excluded due to their limited practical and theoretical experience.

Box 1 **Categorisation of focus group participants.**

Midwifery educators = teachers who are midwives by profession and teach students in midwifery
Midwifery students = midwifery students in their final semester of education
Clinical midwives = midwives in clinical work at hospitals or maternal and child health centres
Midwifery leaders = midwives in leadership positions on clinical wards, or at institutional and regional supervisory levels

In total, 29 participants, whereof midwifery educators (n = 5), midwife clinicians (n = 10), last semester midwifery students (n = 5), and leaders within the midwifery work field (n = 9) were included in the study. The groups consisted of female and male participants, with varied length in current position (Table 1).

**Table 1:** Overview of focus group discussion participants.

Focus groups	Midwifery educators	Midwifery students	Clinical midwives	Midwifery leaders
Number of participants	5	5	10	9
Men	1	1	4	3
Women	4	4	6	6
Number of years in current position	4 to 35 years	–	3 to 10 years	6 months to 16 years

### Instrument and data collection

The qualitative data collection was conducted face-to-face in four FGDs that were led by two lead research team members taking turns on guiding discussion in a safe and sensitive manner. These discussions were based on open-ended semi-structured topic guides that aimed to elicit perspectives and opinions from the participants [16]. The research team members revised two similar but slightly different topic guides that the lead investigator had created, one for the FGD:s with midwifery educators, students, and one for the FGD with the leadership level. They included open-ended, semi-structured questions such as: “As professional midwives, we strive to provide quality midwifery care to all. Let’s start to talk about what are the main barriers for midwives to provide quality midwifery care in The Gambia” and “What are the most important gaps between theory and practice (between education and clinical care)? What is needed for them to be bridged?”. “What is functioning well related to support for quality midwifery care?”.

The focus groups discussions comprised participants from different institutions and were performed separate for each level/cadre. The participants gave oral and written consent to participate in the study, both during phone calls and at the beginning of the recorded FGDs. To be able to collect as much information as possible, a recorder was used with the permission of all participants, to capture the content and nuances of each FGD. The data was then transcribed manually into text and the participants were de-identified, but with cadres kept for each transcript. The researchers and participants were all able to communicate well in English, with no need for a translator. Every focus group gathered once, and the duration of the discussions were 68 to 81 minutes long.

### Data analysis

To analyse data in this empirical qualitative content analysis study, an inductive approach by Elo and Kyngäs [15] was used. Due to the fact that the research area around our purpose is limited, an inductive approach was appropriate, as there was a desire to undertake an in-depth investigation of the research area. Elo and Kyngäs [15] has described three main phases that represent the inductive analysis process. In the first phase, *preparation*, the authors chose what needed to be analysed by transcribing the recorded FGDs. In the second phase, *organizing*, the researcher was acquainted with the information to give them a general understanding of the subject. This was done by reviewing the written transcription multiple times in an objective manner as possible, with the two authors constantly dialoguing their understanding of the data between each other. Then a process known as “open coding” was carried out. According to Elo and Kyngäs [15] this entails that notes and annotations were made adjacent to the text and the content helped to clarify the collected data and better understand the participant’s statements. The authors printed the data, read the text individually and then out loud together while making notes and annotations in the margins of the paper. By writing down key words from different pages without any specific order, they were able to identify various subcategories. The paper with the subcategories was then cut into pieces and arranged in a desired order. This process provided an overview of different preliminary subcategories, some of which could be merged while others formed their own main categories. It was discovered that the data was more intertwined than first presumed, something that finally resulted in the three main headings for the results bringing us into the third phase, *reporting* [15]*,* where the main categories and subcategories were finalized, and the overall result could be interpreted.

### Ethical approval

The participation in the study and sharing opinions were entirely voluntary, with full disclosure of the rights to individual protection, in line with the Belmont Report of ethical principles [17]. All participants signed written informed consent prior to engagement in the study. Ethical approval was granted from The Gambia Government/MRC Joint Ethical Committee, 2023.

## Results

The analysis resulted in three main categories; 1) *The gap between theory and practice, 2) Working in a harsh environment and 3) Facilitating factors that can pave ways forward* described in a total of nine sub-categories, see Table 2. To illustrate the content of the categories, quotes from the FGD are provided within each sub-category, labelled with cadre.

**Table 2:** Main categories and sub-categories of the results.

Main category	Sub-categories
The gap between theory and practice	*National plans and facility-based guidelines*
	*Midwifery education*
	*Becoming a skilled midwife*
Working in a harsh environment	*Scarcity of resources*
	*Encountering community barriers*
	*Midwives - a passionate but demotivated profession*
Facilitating factors that can pave ways forward	*Positive assets for quality midwifery care*
	*Leadership as a tool for a motivated midwifery workforce*
	*Teamwork*

### The gap between theory and practice

#### National plans and facility-based guidelines.

The need for an updated national plan and synchronized guidelines across institutions was emphasized. Leaders expressed their lack of awareness regarding the content of the Midwifery Act, which dates back to 1989 [18], as well as other national plans.

*“Is there a national plan that you are aware of, to safeguard quality midwifery care in The Gambia? - I think it is a national plan to safeguard quality midwifery care in The Gambia, yes there is something in place….” (Midwifery leader)*

The contents of the national plan and facility-based guidelines were not being disseminated effectively, and the information from the national plan was not being adequately filtered down to the midwives providing care. All participants emphasized the importance of keeping knowledge and skills up to date throughout their careers. There was a clear call for increased engagement from policymakers to update and ensure the quality of midwifery care guidelines.

*“(…) people are not updated with new knowledge in regards to midwifery, because sometimes you are aware that this is what we are used to do, this is what we are doing. But that may have changed and that information is not filtered down to the service providers.” (Midwifery educator)*

#### Midwifery education.

Keeping knowledge and skills up to date throughout their working life by applying lifelong learning and continuous in-service training was a described challenge.

*“(…) we have a theory and practice gap. For me I think it’s much bigger - when people graduate from the schools. I think we have a serious problem with the quality of training at institutional level.” (Midwifery leader)*

All participants were aware of the varying lengths of different midwifery education programs. This inconsistency posed a challenge in maintaining the quality of midwifery care and could result in midwives having to perform care measures for which they might not be sufficiently competent, despite holding the same overall title. To ensure students felt adequately prepared for practical clinics, there was a consensus among participants that sufficient materials and access to skills lab activities prior to clinical placements were necessary.

Midwives at practical clinics also needed to provide students with more hands-on experience with different procedures to prevent gaps in their education. Another barrier identified was the presence of too many students at the practice site simultaneously. Additionally, the limitation of students’ hands-on experience was partly due to midwives’ uncertainty about the students’ ability to perform procedures as expected. There was also concern that if a student failed, the supervising midwife might face repercussions.

*“(…) to avoid themselves running into trouble, they (the midwives) try to restrict the students at some point. So, because of that students are not given the opportunity to learn more.” (Midwifery student)*

The students reported gaining practical experience in various clinics, where they received supervision and guidance, effectively bridging the gap between theoretical learning and practical application. They believed that as long as midwifery students made good use of their study time, there was no doubt that skilled midwives would be produced.

#### Becoming a skilled midwife.

The transition from student to independent midwife, where the newly graduated midwife was given the chance to develop over time before being given excessive responsibilities too early, was lacking according to some participants. This was linked to the urgent need in the availability of midwives in the country, especially in the rural areas. This meant that as a recent graduate, you could be appointed as the head of a clinic without prior work experience, which was perceived as highly unsafe.

*For instance, you graduate as an as registered midwife today and then you are posted to a clinic where you are made head of the clinic, and head of the labour ward. Everyone answers to you, and you have never even practised! (midwifery leader)*

In contrast, a leader described an ongoing transition program at selected hospitals in the country, which takes place after the completion of education. Passing a licensing exam organized by the Nursing and Midwifery Council after graduation was seen as a requirement to obtain a license to practice. This examination serves to ensure that midwives have acquired the minimum knowledge and competence necessary to deliver high-quality midwifery care.

*“(...) when we graduate our BSc students, we don’t just send them like that. We have six months’ internship placement at selected hospitals so that it is routine where we monitor them closely. Then from there, after the six months internship you must be certified by the health facility that you were attached to before your training institution writes to the Nursing and Midwifery Council for you to be eligible to sit to the Licensing examination.” (Midwifery educator)*

Lack of access to in-service education for already graduated midwives prevented them from acquiring vital knowledge and much desired certifications as recognitions of their knowledge and skills. The participants expressed the need for on-going education in emergency obstetric care, neonatal emergency care and resuscitation, infection control, and safe birthing practices. They believed that enhancing their knowledge in these areas was crucial.

*“(…) you may not have adequate knowledge needed for that practice. So, if you are in a facility where that might have been the only solution to that woman’s problem in that time, you can’t offer that solution, so it means that there is lot of delays that are going to be involved, because you (…) could not give this help.” (Clinical midwife)*

### Working in a harsh environment

#### Scarcity of resources.

The scarcity of resources, including equipment, mannequins, and ambulances, contributed to the inability to provide quality midwifery care, the majority of the participants claimed. This shortage not only affected the work of midwives but also hampered women’s comfort and led to delays in pregnant women seeking care.

*“When you have all the equipment to provide quality midwifery care, it makes your work easy. We do not always have all we need to provide quality midwifery care.” (Clinical midwife)*

Some of the participants linked the scarcity to the country’s bureaucratic system and the lack of opportunities to generate the money needed for midwifery education - which was originally provided free to all students. Other limitations were obtaining adequate resources for supervised, guided, and supported clinical practices for students; a challenge related to an increasing number of students. *“…there are a lot of students on practice. So that opportunity (to meet the requirements) will be diminished if all of them want to participate or practice at anything.” (Clinical midwife)*

#### Encountering community barriers.

The participants disclosed that some women in The Gambia tended not to prepare for pregnancy, which posed challenges for midwives in delivering quality care.

*“One of the things that affects our care delivery in the Gambia most of the time I would start with the clients we have. Most of them do not know the importance of going for pre-antenatal visit and also antenatal visit.” (Midwifery student)*

Women often delayed registering for maternity care as they desired to keep their pregnancies confidential until it became noticeable. Women in labour tended to delay entering the birth room as much as possible, so they would not have to spend so much time in the facility, resulting in limited opportunities for immediate treatment in case of complications.

*“(…) a woman actually said she will sit home until she knows that she is fully dilated (…). So they will say if you go early, you stay long, so they will stay behind, by the time they already have complications have come in.” (Midwifery student)*

The participants described how the midwives strived to educate and provide guidance to patients on improving their health. Pregnant women nevertheless in some cases prioritized advice from older family members, regarding food taboos, over healthcare professionals’ advice.

*“Some of them believe so much in these food taboos, taboos of like the family adults, so because of that it makes it very difficult the advice they give them in the health facilities” (Midwifery student)*

Among some women, low income prevented them to seek care.

*“There are women who definitely need care but they cannot afford the expense of the care.” (Midwifery student)*

Though the partners support was considered important for the woman’s well-being and truly contributed to an advantage in being able to maintain quality midwifery care, the participants described they rarely accompanied their pregnant woman to the clinic.

*“That social mobilization where the husbands understand the needs for these services. If a woman is pregnant, it needs collaboration from the family. More importantly the husband. The husband’s collaboration is also necessary to ensure quality midwifery care.” (Clinical midwife)*

#### Midwives—A passionate but demotivated profession.

All participants highlighted the shortage of midwives, which stemmed from demotivation despite their passion for the profession, negatively impacting the quality of midwifery care. While midwives’ passion was evident, it was not sufficient to maintain the quality of care in the long run. Recognition from the government and society was necessary. High workloads, long workdays, limited rest, and burnout, coupled with insufficient salaries, led midwives to leave their jobs.

*“At some point you feel like all that tiredness compared to the motivation in terms of finance that you are earning, it doesn’t add up. So, at some point you feel demotivated. You want to leave.” (Midwifery student)*

The lack of employment insurance and sickness allowances was perceived as demotivating for midwives who work diligently. Additionally, workplace bullying in the event of a mistake further discouraged midwives from continuing in the field.

*“(…) it’s sad to say, but the nurses and general midwives are not insured. With all this hard work we do, if you get sick, you are responsible for yourself. Because there is no insurance, there is no motivation, there is a kind of bullying, everything that demotivates people. And then when there are greener pastures somewhere, people leave” (Midwifery student)*

Being assigned to a specific workplace without being involved in the decision-was an additional factor for being disillusioned.

*“You have the choice to reject wherever they take you. Rejecting means you can be rebellious, that you wouldn’t go, that’s what the rejecting would be, but you don’t have a choice. (…) most of the time the Chief Nursing Officer or the Director of Nursing will say “no you have to go there or else your service will be terminated” and you wouldn’t want that”. (Midwifery student)*

### Facilitating factors that can pave ways forward

#### Positive assets for quality midwifery care.

Midwives were entrusted with the responsibility of being women’s advocates and their passionate, solution-oriented and woman-centered care was an asset. Improved infrastructure and expanded healthcare facilities in rural areas made it easier for women to access midwifery care although not always easy for midwives posted far away from the capital.

*“(…) health facilities have increased. We have more facilities and in the periphery like in the villages, there is always a focal person in each village who will be a companion to who ever is in need.” (Midwifery educator)*

Competent midwives in the workforce played a significant role also in conducting group education sessions with women. Supportive policies guided midwives in physiological and complicated births, ensuring the provision of quality midwifery care and especially helpful in rural areas. Equal educational opportunities within the field of midwifery for both women and men were another asset described.

#### Leadership as a tool for a motivated midwifery workforce.

Good leadership, permeating all levels of management, education and care could improve the quality of midwifery care through guidance and support of the midwifery workforce.

*The organization will depend on the leadership. If the leadership is weak, it will come down. It’s the key, they are very essential. Because the leadership organize every aspect of service delivery. (Clinical midwife)*

A number of different aspects were mentioned. Having clean wards was an example of practical leadership that had made it easier to maintain good hygienic standards and had improved the possibilities to provide quality midwifery care. Being looked after by the employer and being valued and recognized as a midwife was highlighted as vital. The existence of formal equal gender opportunities within the field of midwifery even at higher levels and in leadership positions were described.

*“I think the (gender)gap is now being bridged... In almost every part of the country here you have female leaders. You go to a healthcare setting, you have female leaders that are administrators. You go to a labour ward, you find female who are labour-ward-in-charge…Even at the policy level, there are so many women” (Clinical midwife)*

Functioning structures for continuous education and skills-training in life saving skills, boosting of already held skills, and supervision in care of both mother and child, during and after childbirth, were sought for as it could motivate and uphold the provision of quality care, and also be life-saving.

*“We have been trained in school, but if you can have follow-up training in maternal complications, how to identify some of these problems, and also neonatal care. Because we are dealing with two lives. The mother and the unborn baby.” (Clinical middwife)*

The need for clinical career paths was mentioned, and housing and mortgage assistance were other considered beneficial incentives for midwives to remain in the profession; and an area in need of improvements.

*“You spend the salary before even the month has end and you be taking loans you know. And the other major issue is, is the issue of transportation. That is also a BIG problem for us. Not everybody can afford a car. And you can leave your house like 7 a clock and you might get to work 9 a clock.” (Midwifery educator)*

#### Teamwork.

Although the doctor’s decision was ultimately final, it often relied on the suggestions provided by the midwife, with doctors listening to the midwives’ suggestions. It was concluded that the respect for each other’s professions was vital. To enhance teamwork, patience and harmonious collaboration were necessary to guide each other in providing the best care, with the welfare of the woman always at the centre. To achieve harmonious teamwork, it was important to stop blaming each other.

*“Sometimes you will find a midwife who is working very hard, everybody recommends her (…). So just because of one mistake they bully you a lot. So because of that then the person is demoralised, the person is no more encouraged.*

*…So if that is removed, let’s say, the person makes a mistake, you address the mistake in a nice way, and then you see ways forward.” (Midwifery student)*

In case of deviations, witnesses were to inform the responsible manager about the issues to improve and prevent their recurrence. The Ministry of Health could support in enhancing teamwork and ensuring professional boundaries were respected by clarifying the responsibilities of different professions.

## Discussion

This FGD study with midwifery educators, clinicians, students and leaders revealed that the gap between theory and practice in a harsh reality was, combined with a shortage of midwives, and lack of recognition, impacting on the quality of midwifery care. Positive assets such as competent solution oriented midwives, improved infrastructure, leadership and teamwork between doctors and midwives could pave the way for quality midwifery care. Women and men’s formalized equal opportunities to become a midwife and the significance of passion were valuable assets for the quality of care.

The provision of quality midwifery care is influenced by many factors, encompassing both facilitators and barriers [2,8,19–28]. Among these, all participants in the present study highlighted the shortage of midwives, coupled with a high workload and limited availability of resources. This stands out as a significant impediment to delivering adequate care. This situation, which is in line with studies in other contexts [8,19–21] not only compromises the quality of care provided but also contributes to exhaustion and burnout among midwives themselves [8,19,22]. High attrition rates among health care professionals in general, and among professional midwives in particular, in the Gambia [2,13] and elsewhere [23,24], further exacerbate the problem in a vicious circle. This increases the demand for midwifery services and strains resources in educational institutes even more, thereby compromising the quality of midwifery education [24]. Various reasons contribute to midwife attrition, including limited career advancement opportunities, managerial and financial issues, as well as personal fulfillment and support deficiencies [13,23,24]. Moreover, the constrained availability of resources necessary for quality care, combined with economic barriers faced by women, hampers midwives’ ability to fulfill their responsibilities effectively [25,26]. However, amidst these challenges, there are facilitators that can enhance the provision of quality midwifery care. Evidence-based guidelines emphasize the cost-effectiveness and high quality of midwifery care, suggesting definite possibilities for improvement [27,28]. These guidelines underscore fundamental practices in midwifery that are universally applicable and do not require contextualization, promoting consistency and effectiveness in care delivery.

Another central finding was the concerns raised related to the transition from student to independent midwife. When new and un-experienced midwives are to be assigned to rural areas, as head of clinics, without prior work experience, the quality of care and safety are compromised. Addressing issues related to midwife preparedness and support is crucial [29]. Midwives often express feelings of unpreparedness for managerial roles, highlighting the need for comprehensive practical skills and leadership orientation, and continous education [30,31]. Establishing national support frameworks, particularly in regions like sub-Saharan Africa, can aid in new midwives’ adjustment, promote learning opportunities, and foster collaboration between educational institutions and healthcare facilities [27,28,32].

Cultural beliefs and societal attitudes also play a significant role in shaping the quality of midwifery care. In the present study, community barriers were described as impeding quality midwifery care. Strategies to educate communities about the importance of supporting pregnant women and dispelling myths regarding taboo practices are essential for improving care quality [8,28]. In these processes, it is central to adhere to women’s quests for professionals who are able to combine clinical knowledge with skills in communication and cultural competence [28]. This requires a leadership that understands the importance of, and facilitates that, clinical midwives are equipped with sufficient time and space to offer flexible and person-centered care. In settings, as in The Gambia, where the midwifery workforce is signaling risks of resignation and fatigue this is even more pivotal. Addressing work-related health issues and burnout among midwives is imperative for ensuring patient safety and breaking the cycle of exhaustion [33,34]. Creating nurturing environments within healthcare facilities, emphasizing respectful collaboration among healthcare professionals, promoting gender diversity in leadership roles, and ensure that these formal opportunities reaches practice, are also essential for enhancing the quality of midwifery care [35,36]. By prioritizing these facilitators and addressing barriers, healthcare systems, including the health system in The Gambia could create an environment that meets the needs of patients and healthcare providers alike.

### Strengths and limitations

The qualitative methodology employed in this study offers several strengths in elucidating the research purpose. The use of semi-structured questions in face-to-face FGDs allowed for the collection of rich, nuanced data, capturing the thoughts and opinions of the participants effectively [16]. This approach facilitated in-depth discussions and provided opportunities for participants to elaborate on the selected topic, enhancing the depth of the qualitative data collected [14]). Furthermore, the inclusion of four separate FGDs with different cadres mitigated the impact of a limited participant sample, enhancing the transferability of the results to midwifery practice. Qualitative research emphasizes the quality rather than the quantity of data, and the consistent responses across different participant groups suggest a certain level of data saturation, bolstering the validity of the findings [14]. Moreover, the integration of participants’ quotes into the results report enhances the credibility of the study by providing direct evidence of participants’ perspectives [14]. However, certain limitations warrant consideration. Qualitative research, including this study, is susceptible to criticism regarding scientific rigor, particularly concerning method justification, analytical transparency, and the subjective nature of findings. Although efforts were made to reduce bias and enhance rigor, the absence of universally accepted evaluation standards poses challenges for establishing research validity convincingly. Additionally, the analysis focused solely on the manifest content of the transcriptions, potentially overlooking valuable non-verbal cues such as laughter, silence, and posture [15]. While the authors believe that no critical information was missed based on the straightforward responses of participants, the exclusion of non-verbal elements could have limited the depth of understanding.

## Conclusions

Gaps between theory and practice, combined with a shortage of midwives, lack of resources, insufficient recognition and high attrition rates impact the quality of midwifery care in The Gambia. Despite these challenges, midwifery in The Gambia is a trusted profession with potential to ensure quality care and save lives, reaching individual women and their children during pregnancy, birth, and postnatal care. For this potential to be realized and utilized in full, a supportive working environment, strong leadership backing for midwives, and enhanced teamwork are essential components. Guaranteed employment after completing education, equal opportunities for men and women to become midwives, and the passion for women and newborn’s are valuable assets to be carefully maintained within the healthcare system.

Addressing the gaps between theory and practice and strengthening incentives for midwives to remain in their profession and workplace are crucial for improving the quality of midwifery care.

## Supporting information

S1 File Topic guide. (PDF)
