# Supplementary material for: Midwifery care in The Gambia: A focus group study with clinical midwives, midwifery students, educators and leaders on how barriers and facilitators impact quality midwifery care
Source: PLoS One. 2025 Feb 7;20(2):e0318304. doi: 10.1371/journal.pone.0318304 (PMC11805431; doi:10.1371/journal.pone.0318304)
Supplement: S1 File — (PDF) [file pone.0318304.s001.pdf]

Supplementary file 1, Topic guides

Topic guide - midwifery students, educators, clinicians

You are invited to this group discussion as you are a midwifery student, clinical midwife, midwifery educator. This discussion is part of a larger research project that aims to develop, implement and evaluate a model to sustain vital midwifery skills with an attached component also for leaders. Your participation is important as it will contribute to a planned project that will be meaningful and relevant content. In the long run your participation will **contribute to quality maternal and newborn care provided by midwives.**

Socio demographic information

	Age	Gender	Midwifery educator No, Yes, years	Midwifery Student No, Yes, years	Clinical practice as midwife: No, Yes, years	Leadership position: No, yes, years	Current position, and years
1							
2							
3							
4							
5							
6							
7							

A. Providing quality midwifery care

1. As professional midwives, we strive to provide quality midwifery care to all. Let's start to talk about what are the ***main barriers*** for midwives to provide quality midwifery care in The Gambia

2. What are the ***main facilitating factors or resources*** for midwives to provide quality midwifery care in The Gambia?

3. **How are guidelines developed and implemented?**

4. ***Teamwork*** can be a part of the process of providing care. Based on our experience, please describe how this is practiced in the maternity and newborn care.
(Please give examples. Teamwork between whom. Is it important? Why/why not? Could it be improved? How?)

5. ***Midwifery education*** is a base for quality midwifery care. Please let's talk about how this is functioning in The Gambia.
(Does the midwifery education teach quality midwifery care? What is functioning well? Less well? Is there room for improvements, and in such case, how?)

6. Sometimes there is a **gap between theory and practice**. What are your thoughts on this in The Gambia?
   (Are there gaps? What kind? Why? How could they be overcome?)

7. Let's talk about **the role of leadership** and start with **policy level**. To the best of your knowledge, does a policy aimed at supporting the midwifery workforce exist? If so, when was it last reviewed? How would you describe the support you as midwifery workforce in The Gambia get from policy level (this can be Ministry of Health, Units/Directorates, and Regional Directorates of Health Services)
   (What is functioning? What is lacking? Any suggestions on improving leadership and policy? What could be developed?)

   7. On **facility-based leadership and management** level:
   a) How does the leadership at facility level work support the working environment for midwives?
   b) What is functioning well related to support for quality midwifery care?
   c) What is functioning less well? What could be developed?

   8. There is a **professional association** for midwives in The Gambia. What is its role and how does it provide support to professional midwives?
   (What is functioning well? Less well? What could be developed?)

B. Working environment and midwives leaving the workplace or profession

1. When you think of the working conditions and the working environment for midwives in The Gambia, what are your spontaneous thoughts? Please describe.
   (What factors facilitates a good working environment in the Gambia? What could be improved?)

2. Do you think that midwives in The Gambia feel encouraged and enabled to provide quality care in a respectful way? Please describe.

3. A global concern is that midwives leave the workplace, and sometimes also the profession. Why do midwives leave their work positions in The Gambia? (What are the main reasons? Is this a big or small problem?)

4. What could be done to retain more midwives in their work positions in the Gambia?

5. Would a mentorship system contribute to midwives' decisions to leave or stay in the profession? In that case, how?

C. Designing a course in professional development for midwives

1. Are ongoing training programmes (including Continuing Professional Development courses/workshops/seminars) readily made available to midwives? If so, how often? Any suggestions for improvements on ongoing training programmes? (schedule(s) and/or content, please give examples).

2. If you had the possibility to design a course in professional development that focuses on the development of midwifery skills, what would you emphasize, content wise? (please describe, examples...)

3. Would ongoing training in vital midwifery skills contribute to the midwives' decision to leave or stay in the profession? In that case, how?

4. How could the skills gained in such a program be sustained in the long run?

Is there anything more you would like to add?

Thank you so much for your participation!

Topic guide – midwifery leadership and management

- You are invited to this group discussion as you are leader or part of a management linked to midwifery. This discussion is part of a larger research project aimed at developing, implementing and evaluating a model to sustain vital midwifery skills with an attached component also for leaders. Your participation is important as it will contribute to that the planned project will be meaningful and with relevant content. In the long run your participation will **contribute to quality maternal and newborn care provided by midwives.**

Socio demographic information

	**Age**	**Gender**	**Midwifery educator No, Yes, years**	**Clinical practice as midwife: No, Yes, years**	**Leadership position: No, yes, years**	**Current position, and years**
1						
2						
3						
4						
5						
6						
7						

A. National commitment Let's first focus on the policy level and start with the vision, strategic plans and the national/commitment to provide mothers, and children a pathway to a healthy life.

Is there a *national plan* to safeguard quality midwifery care to be provided by a competent midwifery workforce, and is such case, how is it operationalized? Are there any gaps in how this goes from policy to practice, and if so, which are these? How is this overseen?
Is there a commitment from national level, and how solid would you describe it to be? How does it look like? Are there any gaps in this commitment as you see it?
To what extent are the role and function of professional midwives included in the commitment to provide health to women and children?

B. Guidelines is another aspect of ensuring quality care.
Are international and national and local guidelines aligned and followed in education and care? Please describe. Are there any gaps? How are these guidelines developed? How is adherence ensured and how are these guidelines updated?

C. Professional development and skills
At the *clinical institution*, is there a plan to safeguard quality midwifery care to be provided by a competent midwifery workforce, and is such case, how is it operationalized? Are there any gaps in how this is implemented? How is this overseen?
Please describe if there is an *overall national plan* for continuous professional development for the midwifery workforce, and in such case, what are the focus areas? How is this plan being implemented?
Please describe if there is a plan for continuous professional development for the midwifery workforce at *your institution*, and in such case, what are the focus areas? How is this being implemented?

Are sufficient resources provided to ensure that the midwifery workforce is up to date to provide quality midwifery care? Please describe.
Are there any particular areas in need of skills development within the midwifery workforce at your institution, and is there a plan for this? Please describe. (Are there sufficient resources)
How can the skills and knowledge gained in-service trainings and courses in professional development be sustained in the long run? Are there any plans for this at your institution?

D. The midwifery education
What are the most important gaps between theory and practice (between education and clinical care)? What is needed for them to be bridged?
How does the government ensure a functioning chain and alignment between midwifery education and clinical practice? Are there room for improvements here and in such case, how? How is adherence ensured and by whom?
Between your *clinical institution and the educational institution(*s), how is the alignment between what is taught in theory and implemented in practice? Are there room for improvements here and in such case, how? How is this being followed u, and by whom?
(Are sufficient resources provided on *national level* to ensure that the midwifery education can provide midwifery students with the skills needed for future quality midwifery care? Please describe.
Are sufficient resources provided in *your institution* to ensure that the midwifery education can provide midwifery students with the skills needed for future quality midwifery care? Please describe.)

E. The working environment
How is it ensured *at your institution* that the midwifery workforce is safe, secure and able to provide quality midwifery care in a supportive working environment? How is this prioritized? If not, how could it be improved?
What in the working environment can attract midwives to retain in the profession and workplace?
What *national* human resource policies are in place ensuring that the midwifery workforce are safe, secure and able to provide quality midwifery care in a supportive working environment? How is it being implemented? Would you say this is of high priority at policy level, and if not, how could it be improved?

F. Retention of staff
What are the reasons behind professional midwives leaving their work positions in The Gambia? Is this a concern for the *policy level*? Please describe. Is there a *national plan* to retain midwives, and in such case, how is it operationalized?
Is this a concern at *your institution*? Please describe. Is there a facility-based plan to retain midwives, and in such case, how is it operationalized?
Does gender has anything to do with midwives leaving the profession?

G. How should **a course for professional development in vital midwifery skills** be framed to

a. Make it more attractive for midwives to retain at their workplace?
b. Address factual needs (content-wise)?
c. Ensure that the skills are sustained in the long run?

H. **Teamwork and collaboration** is one aspect of ensuring quality care. In what ways and to what extent are teamwork and collaboration between different health professions in maternity care supported *on policy level*? Can you give examples? Could this be improved, and if so, in what ways?

In what ways and to what extent are teamwork and collaboration between different health professions in maternity care supported on *institutional level*? Can you give examples? Could this be improved, and if so, in what ways?

I. How is the p**rofessional association** involved in development of guidelines?

Is there anything more you would like to add?

Thank you so much for your participation!
